# Molecular and Cellular Mechanisms of Spinal Cord Stimulation: Linking Dorsal Horn Circuits, Glia, and ECAP-Guided Therapy

**DOI:** 10.3390/ijms27167373

**Published:** 2026-08-18

**Authors:** Milan Patel, Alison Deng, Ameya Belamkar, Jamal Hasoon, Alan D. Kaye, Alaa Abd-Elsayed

**Affiliations:** 1 New York Institute of Technology, College of Osteopathic Medicine, Arkansas State University, Jonesboro, AR 72401, USA; mpate337@nyit.edu; 2 Stritch School of Medicine, Loyola University Chicago, Maywood, IL 60153, USA; adeng1@luc.edu; 3 School of Medicine, Indiana University, Indianapolis, IN 46202, USA; avbelamk@iu.edu; 4 Department of Anesthesiology, University of Texas Health Science Center at Houston, Houston, TX 77030, USA; jamal.j.hasoon@uth.tmc.edu; 5 Department of Anesthesiology, School of Medicine, Louisiana State University, Shreveport, LA 71103, USA; alan.kaye@lsuhs.edu; 6 Department of Anesthesiology, School of Medicine and Public Health, University of Wisconsin, Madison, WI 53792, USA

**Keywords:** spinal cord stimulation (SCS), tonic SCS, high-frequency SCS, burst SCS, closed-loop SCS, evoke compound action potential (ECAP)

## Abstract

Spinal cord stimulation (SCS) is a widely used neuromodulatory therapy for chronic neuropathic pain, yet the cellular and molecular mechanisms underlying its clinical efficacy remain incompletely understood. This review synthesizes current literature on the neurophysiology of pain transmission and the mechanistic basis of major SCS paradigms (tonic, high-frequency, burst, and closed-loop stimulation), highlighting how each modality engages distinct dorsal horn circuits, glial and inflammatory pathways, as well as supraspinal networks involved in the affective dimension of pain. Particular attention is given to the evoked compound action potential (ECAP) as an emerging electrophysiological biomarker that enables real-time, feedback-guided stimulation and offers insight into the biophysical determinants of dorsal column activation. We also examine preclinical and clinical evidence linking SCS to modulation of central sensitization, neuroinflammatory signaling, and autonomic regulation, while identifying persistent gaps in mechanistic understanding. Finally, we discuss future directions, including AI-assisted, personalized SCS programming and expanding indications beyond classical neuropathic pain, underscoring the need for multimodal experimental approaches to more precisely define how SCS achieves analgesia.

## 1. Introduction

The conceptual foundation of spinal cord stimulation (SCS) is the gate control theory of pain, proposed by Melzack and Wall in 1965, which posits that peripheral pain signals are modulated in a “gate-like” mechanism in the dorsal horn of the spinal cord before the pain experience is transmitted to the central nervous system (CNS) [1]. This theory revolutionized the understanding of pain mechanisms and continues to serve as the physiological basis for neuromodulatory treatments such as SCS [2]. In 1967, Wall and Sweet gave empirical credence to this theory by successfully controlling pain via electrical stimulation of peripheral nerves [3]. Historically, the first SCS was implanted by Shealy et al. via the subarachnoid route to stimulate the dorsal column, effectively “closing the gate” at the substantia gelatinosa to treat intractable lower extremity pain in 1967 [4,5].

Furthermore, there has been growing evidence of the bidirectional communication of cortical and subcortical circuits. Thus, not only is nociceptive transmission affected by neuromodulation, but also higher-order functions such as emotion and cognition are also involved. As a result, spinal cord stimulation, while highly effective for nociceptive modulation, can also play a role in affecting cortical-subcortical bidirectionality [6].

Over the subsequent decades, SCS technology evolved significantly. Related to complications with the subarachnoid route, there was a transition to the epidural route throughout the 1970s. Throughout the 1970s and 1980s, percutaneous and paddle-type leads were developed, and externally powered and fully implantable pulse generators were introduced. In the 1990s and 2000s, multichannel, programmable devices, rechargeable batteries, and more sophisticated electrode arrays were developed, improving the efficacy and safety profiles of SCSs. Recent advances have introduced high-frequency, burst, and closed-loop stimulation paradigms, MRI-conditional devices, and remote programming capabilities for more personalized pain control [7]. There has been an expansion in the number of United States Food and Drug Administration (FDA)-approved indications in past decades, and there has been tremendous growth in the use of SCS for chronic neuropathic pain in recent years, with 35,000 devices being implanted annually, representing an estimated market valuation of $2 billion [7,8,9].

While the present review focuses on the use of SCS for peripheral pain, SCS has established and emerging applications across a range of other conditions. Within pain management, these include refractory angina pectoris, ischemic limb pain in peripheral vascular disease, intractable visceral pain, and cancer-related pain syndromes such as chemotherapy-induced peripheral neuropathy [10,11,12,13,14,15]. Beyond pain, SCS is under active investigation for motor and autonomic recovery after spinal cord injury, gait impairment in Parkinson’s disease, and autonomic modulation in heart failure [16,17,18,19,20,21,22,23,24]. A comprehensive review of these indications is beyond the scope of the present work.

Despite demonstrating clinical efficacy in the treatment of refractory neuropathic pain disorders [25,26], the mechanism by which SCS achieves these effects is not fully understood. This review, therefore, summarizes our current understanding of and highlights gaps in knowledge in the physiological mechanisms of SCS.

### Literature Search

This comprehensive narrative review was used to find evidence involving molecu-lar, cellular, neurophysiological, and clinical mechanisms of spinal cord stimulation. PubMed was searched for full-text, peer-reviewed articles from the dates of January 2010 through July 2026. The search terms included were “spinal cord stimulation” with “mechanism of action,” “pain modulation,” “gate control,” “dorsal horn,” “supraspinal mechanisms,” “neurotransmitters,” glial cells,” and “neuroinflammation.” The studies selected included human clinical trials, preclinical animal studies, randomized controlled trials, observational studies, systematic reviews, meta-analyses, narrative reviews, and case series. For exclusion criteria, we did not include duplicate publications, individual case reports, conference abstracts, editorials, commentaries, letters to the editor, and publications.

## 2. Basic Neurophysiology of Pain and the Spinal Cord

Melzack and Wall’s gate control theory of pain revolutionized our understanding of the neurophysiological basis of pain mechanisms [1,2,27]. In their original manuscript, Melzack and Wall laid out two central tenets underlying the neuromodulation of pain transmission [1]. First, they asserted that stimulation of large, non-nociceptive Aβ-fibers can “close” the metaphorical gate via a feedforward mechanism by stimulating inhibitory interneurons, thereby suppressing the transmission of pain signals to higher centers. Conversely, stimulation of small-diameter nociceptive fibers “opens” the gate by inhibiting these interneurons and facilitating pain transmission. The interplay between these excitatory and inhibitory influences ultimately determines pain perception [28]. Thus, this theory fundamentally shifted the understanding of pain from a simple, direct transmission of nociceptive signals to the brain to a dynamic process involving modulation at the level of the spinal cord and higher levels. Since this theory was published, further research has elucidated much greater complexity in pain mechanisms, including critical roles of neurochemical modulation, glial and inflammatory modulation, and supraspinal input [29].

The sensory pathway of pain begins at the peripheral terminals of primary afferent neurons, which transmit nociceptive information from the periphery to the dorsal horn of the spinal cord. There are three main types of afferent neurons in the peripheral nervous system (PNS): Aβ-fibers, Aδ-fibers, and C-fibers. Each fiber type has unique structural properties that make it suited for the sensation and transmission of different sensations. Aβ-fibers are large, heavily myelinated, and have low activation potential; these fibers can quickly transmit action potentials, making them suitable for the sensation of light touch and transmitting tactile information. Aδ-fibers are smaller, less myelinated, have a greater activation potential, and are responsible for responding to thermal and mechanical stimuli. C-fibers are the smallest and are unmyelinated, making them the slowest conducting afferent fibers. They have the greatest threshold for activation and thus selectively detect “painful” stimuli or nociception. Aδ-fibers and C-fibers can be called “nociceptors” as they respond to noxious mechanical, thermal, or electrical stimuli [30].

Most nociceptive Aδ and C-fibers terminate in laminae I-II of the dorsal horn. Within the dorsal horn of the spinal cord, the intensity of a noxious stimulus is encoded by both nociceptive-specific (NS) neurons and wide dynamic range (WDR) neurons [26]. NS cells are mostly found superficially and synapse only with Aδ- and C-fibers, firing action potentials upon painful stimuli. WDR neurons, located deeper, receive input from all three sensory fiber types and respond to the full range of stimulation, ranging from light touch to noxious stimuli [30].

Ascending pathways convey this information via spinal projection neurons to various regions in the brain, including the thalamus, periaqueductal gray (PAG), parabrachial nucleus (PBN), and rostral ventromedial medulla (RVM). Many of these projection neurons are located in lamina I, and 80% of these cells express the neurokinin-1 (NK1) receptor for substance P, a neuropeptide released by nociceptive afferent neurons [31]. Additionally, many projection neurons are found deeper in the dorsal horn from lamina III–VI and project predominantly to the thalamus, comprising a significant proportion of the spinothalamic tract. This ascending, primarily sensory pathway provides the sensory component of the pain experience [30].

The thalamus is a relay point for the higher-level processing of nociceptive information. From the thalamus, nociceptive information is sent to various cortical regions comprising the “pain matrix,” which includes the primary and secondary somatosensory, insular, anterior cingulate, and prefrontal cortices [32].

In addition to these ascending pathways, descending pathways, which may be facilitatory or inhibitory, play a critical role in modulating pain sensation [28,33]. Descending inhibitory pathways suppress the transmission of nociceptive information to higher brain centers by reducing the release of pronociceptive mediators from primary afferent fibers, directly suppressing the activity of pain-transmitting neurons, or activating local inhibitory interneurons in the spinal cord. This system is crucial for adaptive pain modulation, such as stress-induced analgesia, by dampening excessive pain signals [27,34].

Descending facilitatory pathways enhance pain by increasing the excitability of spinal pain-transmitting neurons and interfering with local inhibitory mechanisms, thereby amplifying pain perception, and may play a role in phenomena like hyperalgesia and allodynia [35,36,37,38]. The PAG plays a pivotal role in the modulation of pain sensation. It receives inputs from the PBN, hypothalamus, nucleus tractus solitarius (NTS), and various corticolimbic structures, including the amygdala [25,39,40].

The hypothalamus, PBN, and NTS are all involved in integrating autonomic and somatosensory information and are linked with higher-level limbic and cortical structures. The RVM is a heterogeneous region that provides both descending facilitation and inhibition, and its descending pathways are primarily modified by afferents from the PAG, PBN, and NTS. The RVM has “OFF” cells that are excited by opioids and inhibited by nociception and participate in descending inhibition, as well as “ON” cells that are inhibited by opioids and excited by nociception and participate in descending facilitation [28]. Furthermore, recent evidence suggests that ascending and descending pathways may be linked in the modulation of nociception. Tobaldini et al. demonstrated that noxious stimulation triggers an ascending–descending pain modulation pathway that links the mesolimbic system to the PAG–RVM descending system, which may be of adaptive significance to mediate pain-induced analgesia in defensive situations [36].

## 3. Molecular Mechanisms of Pain

Glutamate is the chief neurotransmitter of nociceptive primary afferents and mediates the excitatory neurotransmission between the primary afferent fibers and second-order spinal sensory neurons [25,39]. Glutamate is loaded into synaptic vesicles at the axon terminals of Aδ and C fibers by vesicular glutamate transporter 2 (VGLUT2). Glutamate released at the synapse acts on glutamate receptors, which may be ionotropic or metabotropic. Ionotropic glutamate receptors may be classified into three groups: N-methyl-d-aspartate (NMDA), α-amino-3-hydroxy-5-methyl-4-isoxazole propionic acid (AMPA), and kainate receptors, but kainate receptors make a minimal contribution to synaptic transmission between nociceptive primary afferents and spinal neurons.

Metabotropic glutamate receptors (mGluRs) are G-protein-coupled receptors (GPCRs) that modulate the effects of glutamate transmission at the synapse. Eight subtypes (mGluR 1–8) are divided into three groups with varying synaptic localizations and thus differing effects on glutamatergic transmission. Excitatory amino acid transporters (EAATs), localized to the cell membranes of neurons and glial cells, are responsible for glutamate clearance from the synaptic cleft [41]. EAATs ensure low basal glutamate concentration in the synaptic cleft and are protective against glutamate excitotoxicity.

When evoked by peripheral inflammation, neuropathy, or pruritogens, primary afferents may concurrently release neuropeptides, neurotrophins, or endorphins with glutamate that modulate glutamatergic neurotransmission. In contrast to synaptic glutamatergic signaling, these molecules are released extrasynaptically and may induce excitation, disinhibition, or inhibition of spinal neurons via volume transmission. Neuropeptide receptors are GPCRs that regulate protein phosphorylation and gene expression; thus, neuropeptides can influence the biophysical parameters of postsynaptic neurons and alter the sensitization of specific ion channels. Although not working in direct concert with glutamate, in this manner, neuropeptides can indirectly modulate glutamate-evoked postsynaptic responses. As such, nociception involves a complex interplay between glutamatergic synaptic transmission and nonglutamatergic extrasynaptic volume transmission [41].

Additionally, several factors influence the presynaptic modulation of pain. Activation of metabotropic glutamate receptors causes presynaptic inhibition either via coupling to G_i/o_ proteins in the case of mGluR2, mGluR3, mGluR4, and mGluR7 or via G_q/11_ protein-induced activation of the retrograde endocannabinoid system in the case of mGluR5. The retrograde endocannabinoid system attenuates glutamate release via binding its endogenous ligand, 2-arachidonoyl glycerol (2-AG), to the type 1 cannabinoid receptor (CB1R) [41].

The release of glutamate, neuropeptides, and neurotrophins from primary afferents activates a variety of neurons in the superficial spinal dorsal horn, including inhibitory neurons. These inhibitory neurons may project back to nociceptive afferent neurons and release the neurotransmitters gamma aminobutyric acid (GABA) or glycine via axo-axonic contacts. The binding of the endogenous opioid ligands enkephalin, dynorphin, and endorphin to opioid receptors, as well as the binding of nociception/orphanin FQ (N/OFQ) peptide to the N/OFQ receptor, are known to cause presynaptic inhibition of nociceptive transmission. Presynaptic facilitation may be mediated by the purinergic system using adenosine 5′-triphosphate (ATP) as an excitatory neurotransmitter. Presynaptic modulation is further controlled by descending monoaminergic fibers from supraspinal nuclei. The release of norepinephrine, dopamine, and serotonin from these descending fibers has various effects on nociception depending on the specific receptor subtype that is activated [41].

Glial cells and inflammation play a central role in developing and maintaining neuropathic pain states. Nerve injury in the CNS causes the affected tissue to release chemokines and inflammatory mediators that rapidly induce local microglial cells to release pro-inflammatory cytokines, such as interleukin-1β (IL-1β), interleukin-6 (IL-6), and tumor necrosis factor alpha (TNF-α) [39,42,43]. These pro-inflammatory cytokines increase the excitability of primary afferent neurons, thus facilitating pain signaling [44]. In the normal state, the subsequent release of anti-inflammatory cytokines can restore the balance between pro- and anti-inflammatory molecules. However, a failure to maintain this balance may cause hyperalgesia and allodynia and ultimately the development of chronic neuropathic pain [39,45].

The activation of microglial cells peaks 4–7 days following neuronal injury, suggesting microglia play an integral role in the onset of neuropathic pain. These microglial cells may activate neighboring astrocytes, which reach a peak activation during the second week following injury, suggesting astrocytes play a role in both the onset and maintenance of neuropathic pain [44]. Activated astrocytes play a key role in developing neuropathic pain through several mechanisms, such as releasing proinflammatory cytokines, regulating receptors like Toll-like receptor-4 (TLR-4), and enhancing Ca^2+^ transmission [39]. There is accumulating preclinical evidence suggesting that using SCS paradigms utilizing high-frequency stimulation is more effective in restoring the central inflammatory balance and thus alleviating neuropathic pain [39].

A key concept underpinning the development of chronic pain is central sensitization, which refers to an increase in responsiveness of the nociceptive circuitry of the CNS due to increased excitability and synaptic efficacy, as well as reduced inhibition of the pathway [46]. Central sensitization is an example of maladaptive synaptic plasticity, wherein the activity-dependent synaptic changes in the nociceptive pathway lead to long-term potentiation (LTP) that persists beyond the initial noxious stimulus [46,47]. These changes are mediated by increased glutamatergic transmission, upregulation of NMDA and AMPA receptors, modulation of ion channels, and activation of glial cells, along with the release of proinflammatory cytokines, all of which maintain the sensitized state [26,46]. Consequently, central sensitization is essential in inducing hyperalgesia and allodynia [48]. As seen in Table 1, a summarization of the various pain mechanisms discussed in this section is laid out with their primary roles listed for comparison.

## 4. Technological Advances and Their Mechanistic Implications

### 4.1. Tonic Spinal Cord Stimulation

Tonic SCS is the conventional form of spinal cord stimulation and remains the paradigm most closely aligned with the original gate control theory of pain. In tonic SCS, a low-frequency pulse, usually 10–500 Hz, is delivered at a constant interval, usually 30–500 μsec. These pulses create a tingling “pins and needles” sensation known as paresthesia that masks the underlying pain sensation. It is well-studied and well-supported in the treatment of chronic neuropathic pain [49]. Tonic SCS is based on the gate control theory of pain. The central idea is to electrically stimulate the dorsal horn of the spinal cord, which contains large Aβ fibers, to close the gate to pain transmission and provide pain relief [5]. While tonic SCS is the most supported and used form of SCS, there are notable limitations. First, tonic SCS has only been shown to reduce pain by ≥50% in 50–70% of patients with various chronic pain disorders, with an average reduction in pain of 50–60%. Furthermore, tonic SCS has less efficacy in areas that are difficult to reach, such as the extremities or groin [50].

Additionally, the paresthesias induced by tonic SCS are susceptible to postural variation due to changes in distance between the implanted lead and spinal cord, which can lead to patient discomfort or overstimulation [51]. Lastly, there is the potential for habituation, where patients will experience decreased pain relief from tonic SCS and require new waveforms [52]. Table 2 dives into the various targets of tonic spinal cord stimulation as well as the roles in which they operate. There is increasing support for other innovative SCS methods, including high-frequency, burst, and closed-loop SCS, that attempt to address these limitations [53,54].

### 4.2. High-Frequency Spinal Cord Stimulation (10 kHz)

A recent innovation in the field of SCS is the development of the high-frequency stimulation technique, delivering waveforms at 10 kHz [55]. Unlike conventional tonic SCS, high-frequency SCS provides analgesia without producing paresthesia, which has been shown to be preferred over paresthesia-inducing modalities [56]. The molecular mechanism of high-frequency SCS remains unclear, but likely extends beyond classical gate-control theory. Rather than rely on Aβ fiber gating, high-frequency SCS activates inhibitory interneurons in the dorsal horn [57]. Table 3 provides additional information on Aβ fiber gating and inhibitory interneurons in the dorsal horn. However, based on computational modeling, several hypotheses suggest that high-frequency SCS may provide relief through modulation of medium and small fibers, interference with depolarization signals, and inhibition of protein kinases [58,59]. Therefore, the large-diameter axons that carry the vibratory sensation are blocked. Follow-up studies are required to elucidate its mechanism fully.

Two pivotal RCTs have established the clinical efficacy of high-frequency SCS for neuropathic pain. The SENZA-RCT enrolled 198 patients with chronic back and leg pain and compared high-frequency SCS to traditional low-frequency SCS. At 24 months, high-frequency SCS demonstrated statistically significant superiority, with responder rates of 76.5% versus 49.3% for back pain and 72.9% versus 49.3% for leg pain (*p* < 0.001) [60,61]. Notably, pain relief was achieved in the absence of paresthesias. The SENZA-PDN trial, the largest SCS RCT in painful diabetic randomized 216 patients to 10 kHz SCS plus conventional medical management (CMM) or CMM alone [62]. At 6 months, 79% of patients in the SCS group achieved the primary endpoint of ≥50% pain relief without neurological worsening, compared with 5% in the control group (*p* < 0.001). Durability was confirmed at 24 months, with 90.1% of patients maintaining ≥50% pain relief [63].

### 4.3. Burst Spinal Cord Stimulation

Burst SCS was developed to better mimic the natural burst firing patterns of neurons and appears to engage both the sensory and affective dimensions of pain. Burst SCS is a paradigm in which SCS is delivered in clusters, typically in groups of five pulses at 500 Hz [64]. It was designed to mimic the natural firing of neurons. In particular, thalamic cells can transfer messages in tonic and burst models. However, burst neuron firing more strongly activates cortical neurons than tonic firing [65]. As seen in Table 4, the primary tagret and mechanistic role of key burst stimulation factors is summarized below. Thus, burst SCS was applied to the spinal cord and effectively relieved pain, including back and limb pain [66,67].

Additionally, burst SCS does not cause paresthesia in patients, providing an additional benefit. Furthermore, compared to tonic SCS, burst SCS is also suggested to affect the medial spinal-thalamo-cortical pathways, which are involved in pain’s affective and emotional processing [68]. Previously, researchers have found evidence that burst-SCS drives supraspinal modulation and cortical-level neuroplasticity changes [69]. These findings suggest that the therapeutic effect extends beyond segmental gate-control modulation, influencing both the medial (affective-emotional) and lateral (sensory-discriminative) pain pathways through supraspinal engagement. Burst SCS works on dual pathways in pain’s physiological and emotional aspects, while tonic SCS focuses on the physiological. Studies have supported this mechanism by finding that patients with the burst modality experienced greater psychometric improvement [69].

### 4.4. Closed-Loop Spinal Cord Stimulation

**Figure 1:**
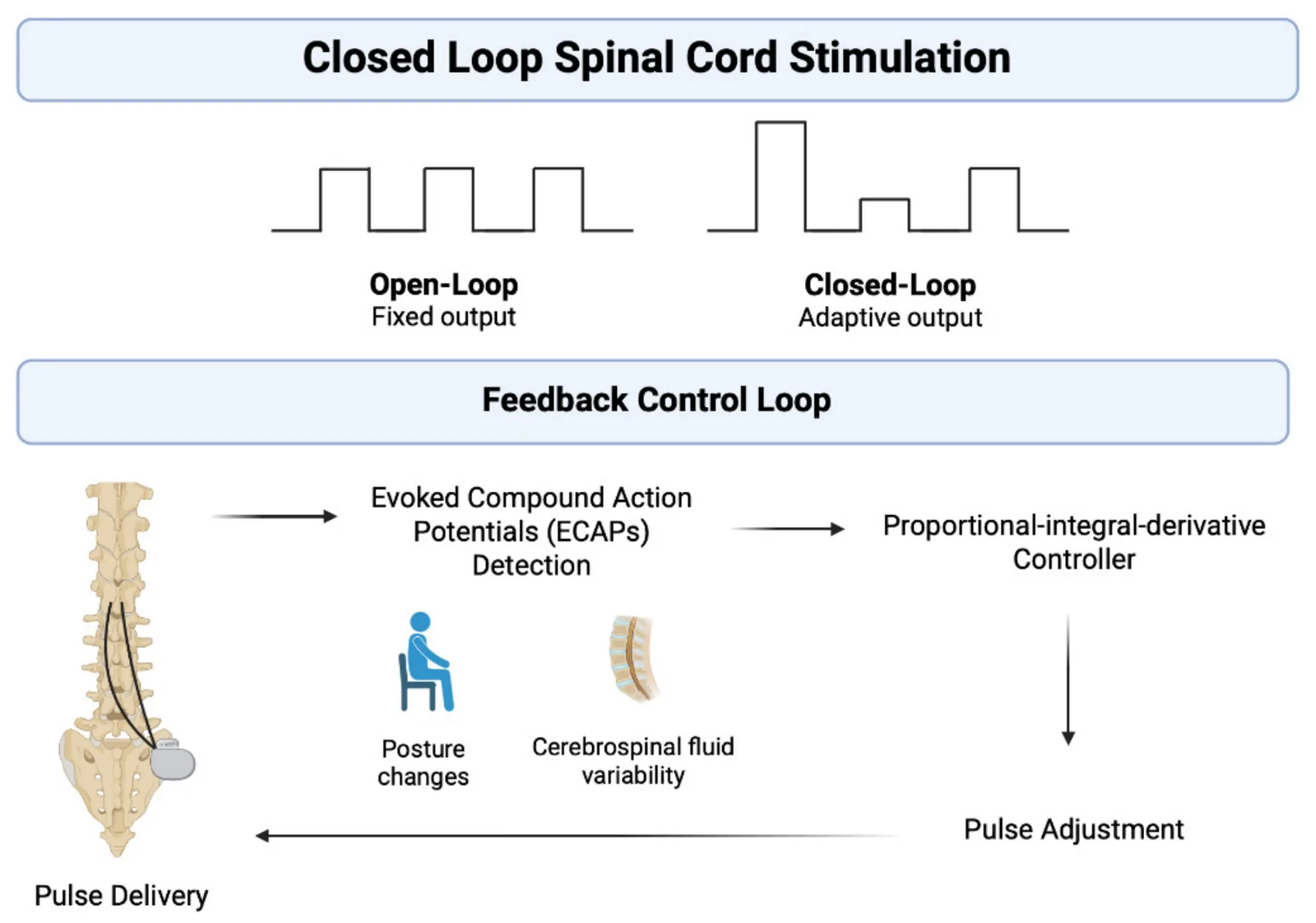
Schematic outlining closed-loop spinal cord stimulation with proportional-integral-derivative controller-mediated pulse adjustments. Created in Biorender. Deng, A. (2026) https://app.biorender.com/citation/6a4aa9d9f473d61d541dd1ba, accessed on 6 July 2026.

Unlike the preceding paradigms, closed-loop SCS represents an adaptive control strategy rather than a novel stimulation waveform. Instead of introducing a different biological mechanism, it continuously adjusts stimulation output using ECAP feedback to maintain consistent neural activation despite physiologic changes [70]. Rather than a new waveform like high-frequency or burst, closed-loop SCS represents a new control system. Figure 1 describes the overall pathway which we will go into throughout this section. The closed-loop system mitigates the effects of habituation and variability associated with traditional tonic SCS by delivering a consistent tissue activation [52]. A 2023 systematic review found that closed-loop SCS was recommended for back and leg pain over traditional, open-loop SCS [71]. Closed-loop systems deliver information through evoked compound action potentials (ECAPs), a quantitative measure of dorsal column activation representing the summation of action potentials [72]. The molecular basis for using ECAPs in spinal cord stimulation is the synchronous depolarization of Aβ fibers in the dorsal columns following electrical stimulation. These factors and others can be found in Table 5 for additional information and clarification. When an epidural electrode delivers a pulse, it induces membrane depolarization in these axons, generating action potentials that propagate along the fiber. The ECAP is the extracellular summation of these action potentials from multiple recruited fibers [73,74,75]. This process is governed by the biophysical properties of the axonal membrane, including voltage-gated sodium and potassium channels, and the spatial distribution of the electric field. SCS preferentially activates large-diameter, myelinated fibers due to their lower activation threshold and proximity to the electrode [73,76]. The amplitude and morphology of the ECAP reflect the number and synchrony of recruited axons, which is modulated by stimulation parameters (amplitude, pulse width, frequency) and anatomical factors (electrode position, cerebrospinal fluid thickness) [77,78]. Thus, ECAPs represent the neural activation of the dorsal columns, and this may be used as input to a feedback loop such that the SCS delivery can be modified to maintain the neural response within a target window for improved consistency in therapeutic delivery.

There are a number of factors that can affect the degree of neural activation provided by open-loop SCS devices which may be accounted for by ECAP feedback regulation in closed-loop devices. Periprosthetic fibrosis increases the resistance of the tissue surrounding the electrode, which can elevate the activation threshold or neurons and reduce the efficacy of stimulation. Other changes in impedance dynamics, including changes due to tissue encapsulation, fluid shifts, or electrode-tissue interface alterations, also affect the amount of current delivered to neural targets [79]. Postural shifts alter the spatial relationship between the spinal cord and the epidural electrode and change the thickness of the cerebrospinal fluid (CSF) layer, which can cause substantial fluctuations in neural activation [80]. Even small changes in spinal cord position, such as during the cardiorespiratory cycle, can have major impacts on the amount of current reaching the spinal cord because the density of current during spinal cord stimulation decreases with the square of the distance between the electrode and the spinal cord. Consequently, at a fixed stimulation output, the extent of spinal cord activation can vary substantially [74].

To overcome these limitations, the closed-loop system employs a proportional-integral-derivative controller that continuously minimizes the difference between the measured mean ECAP amplitude and the target ECAP amplitude target by automatically adjusting the therapeutic current amplitude in real time for every stimulus. This is a frequency dependent process and can occur more than 100 times per second, thereby allowing the system to maintain a stable neural response in which the average error between the target and measured ECAP amplitudes is effectively zero. For example, a 50 Hz device will make 50 readings of the ECAP per second, totaling over 4 million adjustments per day to maintain a constant ECAP amplitude [74].

However, variations in the measurement of ECAP can limit the ability of closed-loop SCS devices to provide consistent neural activation. Postural shifts can cause significant fluctuations in ECAP threshold and amplitude because the ECAP is influenced by both the motion of the spinal cord relative to the stimulating electrode as well as the recording electrodes [81]. A study by Brucker-Hahn et al. highlights the challenges in providing consistent neural activation utilizing ECAP feedback. Combining empirical data and computational modeling, this study found that approximately twice as many fibers were activated in a prone vs. supine position for a given ECAP amplitude. Furthermore, the study demonstrated that maintaining a constant ECAP amplitude does not necessarily ensure consistent neural recruitment within the spinal cord, highlighting a potential shortcoming of the closed-loop SCS design that may need to be optimized with further research [82]. Table 6 and Table 7 compare the results seen in all of the neuromodulatory paradigms that were discussed as well as key differentiating information.

## 5. Experimental Models and Biomarkers

### 5.1. Animal Models Used to Explore Spinal Cord Stimulation Mechanisms

Animal and preclinical models provide excellent introductions for initial exploration efforts to understand the underlying mechanisms in spinal cord stimulation. Various animal models have utilized a variety of differences in apparatus setup in the form of lead placement and stimulation parameters.

In a systematic review by Mugan et al., 78 animal model studies were included, with 46 displaying excellent efficacy. This review analyzed and assessed pain models focused on complex regional pain syndrome II, persistent spinal pain syndrome II, and nontraumatic peripheral neuropathies [83]. When assessing CRPS, PSPS, and non-traumatic peripheral neuropathies in humans, we see elevated levels of IL-1β and IL-6 in human CSF. However, with spinal cord stimulation therapies, we can see these levels decreased with therapeutic relief being felt by the patient [84]. In the previous sections of this review, the mechanisms utilized by various spinal cord stimulation modalities were explained as needed.

Animal models, however, serve a significant purpose in helping elucidate the exact function of neuromodulatory techniques. In 1911, Allen created the first animal SCI models through weight drops, which affected the dorsal spinal cord. Furthermore, SCI and SCS models have been tested in situations involving contusive, compressive, tractive, photochemical-induced, inflammatory injury, and ischemia-reperfusion. However, challenges and issues persist, which warrant further research and testing in animal models. These include the need for more anatomical and physiological correlation between experimental animal models and clinical studies, congruent SCI pathology between species and strains, and additional interpretations of animal study results to inform clinical outcomes in humans further [85].

At the molecular level, animal studies have identified a rich array of mediators underlying SCS analgesia, including increased dorsal horn GABA release that suppresses excitatory amino acid transmission via GABA-B receptor mechanisms, activation of endocannabinoid CB1 receptors, frequency-dependent engagement of µ- and δ-opioid receptors, and cholinergic signaling through muscarinic M4 receptors [86,87]. SCS also activates descending serotonergic pathways from the RVM, with 5-HT2A, 5-HT3, and 5-HT4 receptor subtypes contributing to pain relief, the latter operating partly through spinal GABAergic interneurons [88,89]. At the glial level, neuropathic pain drives microglial and astrocytic activation in the dorsal horn, and SCS has been shown to reduce glial immunoreactivity and attenuate neurotoxic A1-like astrocyte polarization via suppression of the Ca2+/calcineurin/NFAT4 signaling axis, decreasing proinflammatory cytokine release [90,91]. Differential targeted multiplexed programming (DTMP) approaches appear particularly effective at modulating neuron- and glia-specific transcriptomes toward expression levels found in healthy animals, suggesting that stimulation parameters differentially affect neuroglial interactions [83,92].

Despite these contributions, several important limitations of preclinical SCS models warrant consideration. Most animal studies have relied on monopolar electrodes and standardized stimulation parameters (typically 50 Hz, 200 µs, at ~72% of motor threshold), which diverge substantially from the multicontact paddle or percutaneous leads and diverse programming strategies used clinically [83]. The small anatomic scale of rodent models precludes the use of clinical-grade hardware, and computational modeling has demonstrated that per unit current, peak electric fields in the rat dorsal column are approximately 17 times higher than clinical values, with markedly different spatial decay profiles across species, thereby making direct parameter translation between animals and humans intractable [93]. Furthermore, much of the electrophysiological and neurochemical data underpinning mechanistic understanding has been obtained in anesthetized animals, which may confound the very GABAergic and descending modulatory pathways SCS is thought to engage [94]. Behavioral outcome measures also present challenges, as reflex-based assessments such as paw withdrawal thresholds capture hypersensitivity rather than the affective or spontaneous dimensions of pain that drive patients to seek SCS therapy [94].

These methodological constraints help explain why promising preclinical biomarkers (including GABA, serotonin, adenosine, and inflammatory cytokines such as IL-1β and IL-6) have not yet been translated into validated clinical biomarkers or reliable predictors of treatment response. Different SCS paradigms, as mentioned and explained in Table 8, appear to exert distinct effects on the central inflammatory balance, but the quality of available preclinical evidence limits confident interpretation [39].

Future studies should prioritize awake electrophysiological recordings, larger animal models accommodating clinical-grade hardware, outcome measures that capture pain affect, and systematic efforts to validate preclinical molecular findings.

### 5.2. ECAP as a Functional Electrophysiological Biomarker

Biomarkers help determine the disease’s overall pathophysiology and the efficacy of a treatment option. By providing a direct, objective, real-time measure of dorsal column Aβ fiber activation, ECAP can be viewed as a functional electrophysiological biomarker for SCS efficacy [76]. The EVOKE double-blinded randomized controlled trial demonstrates that ECAP-controlled closed-loop SCS provides sustained, durable pain relief and superior holistic treatment response through 36 months compared to open-loop SCS. Specifically, a greater proportion of patients with closed-loop SCS achieved ≥50% pain relief (77.6% vs. 49.3%), ≥80% pain relief (49.3% vs. 31.3%,) and holistic treatment response (44.8% vs. 28.4%). Furthermore, there was greater neural activation and increased accuracy of spinal cord activation in closed-loop SCS. Closed-loop SCS maintained spinal cord activation within the therapeutic window approximately 94% of the time, compared to only 46% for open-loop SCS, paralleling the superior reductions in pain [74].

Furthermore, Mekhail et al. evaluated neurophysiological outcomes in a cohort of patients randomized to closed-loop SCS in the EVOKE trial. They found that, through 36 months, the loss of therapeutic effectiveness was not a failure mode for ECAP-controlled closed-loop SCS, calling into question the notion that tolerance or habituation leads to loss of effectiveness of SCS and providing objective physiological evidence that SCS can provide durable, long-term improvements in chronic pain [74]. Muller et al. analyzed over 600 SCS patients and demonstrated the first evidence of a dose–response relationship in SCS using physiological closed-loop control ECAP-controlled technology. Higher stimulation dose, more time spent above the neural activation threshold, and greater accuracy of therapy delivery were all significantly associated with better pain relief outcomes [95]. ECAP-based closed-loop control improves the precision, durability, and dose-responsive efficacy of SCS relative to open-loop systems and therefore may serve as a potential avenue for more individualized, effective SCS therapy.

## 6. Limitations in Current Understanding

The mechanism of action of SCS remains unclear, particularly regarding high-frequency SCS [64]. Therefore, it would be beneficial to elucidate the mechanism further to comprehend the paradigm fully. There are a few hypotheses regarding its mechanism that remain to be confirmed. Emerging work has centered around themes of suppressing dorsal horn hyperexcitability, modulation of neuroimmune signaling, and indirect engagement of descending inhibitory pathways through supraspinal feedback loops [96,97,98]. Related to differing hypotheses in our scientific literature, further studies are required to verify the plausibility of each hypothesis.

Future studies capable of discriminating among these mechanisms will require multimodal experimental designs including electrophysiology, molecular, and cellular experiments. Recording dorsal horn unit activity can differentiate between blocking signals at the spinal or inhibitory pathways from the brain [99]. Dorsal horn electrophysiological recordings, including WDR reflecting excitability and local field potentials (LFPs) indexing network-level activity could clarify whether analgesic effects arise predominantly from attenuation of spinal hyperexcitability or recruitment of descending inhibitory networks [100,101]. Cerebrospinal fluid (CSF) cytokine profiling and microglial activation markers could test a neuroimmune hypothesis [102]. Parallel assessment of CSF cytokine profiles, along with immunohistochemical quantification of microglial (Iba1, CD68) and astrocytic (GFAP) activation, would elucidate neuroimmune contributions [103]. Moreover, molecular assays measuring expression of biomarkers phospho-NR2B, KCC2, GAD65/67, and GLT-1 would provide quantitative indications of excitatory-inhibitory balance following high-frequency SCS [104,105,106,107].

Growing evidence indicates that the effects of SCS extend beyond somatosensory modulation and include meaningful interactions with the autonomic nervous system (ANS). By influencing dorsal column and dorsal horn pathways, which interact with sympathetic and parasympathetic regulation, SCS can alter cardiovascular tone, stress response, and homeostasis [102]. Recent work suggests that non-invasive brain stimulation can shift the autonomic balance through bidirectional signaling between cortical control centers and autonomic output [108]. SCS mediates nociception and may restore autonomic stability. Further supporting this autonomic framework, emerging evidence indicates that neuroendocrine and stress-related mechanisms act as mediators of neuromodulatory responses. One study showed that physical activity and stress exposure modulate autonomic and neuroendocrine balance through adaptive changes in central regulatory circuits [108]. These findings parallel the effects observed with SCS, attenuating stress-driven amplification of nociceptive processing.

However, it is worth noting that isolating these mechanisms in vivo is difficult. There is no perfect translation from in vitro to the human system, as body systems are complex and have many interacting components. The effects of SCS are delivered by multiple different processes, not a single mechanism, so it is difficult to isolate a single cause and effect [109]. For example, burst SCS is theorized to have a therapeutic effect through physiologic and emotional regulation mechanisms [68]. When testing burst SCS, it would not be easy to parse out the effects based on each mechanism of action.

In addition to difficulty conducting additional experiments in vivo, researchers also have challenges completing validated studies using individual responses. These responses are prone to inter-individual variability and have a level of subjectivity that can affect the rigor of a research study. Pain is subjective and varies by person and diagnosis. It can also be complicated by multiple sites causing pain. Variability is introduced when self-reported data primarily assesses pain [50].

## 7. Future Directions and Innovations

Especially with the advent of closed-loop SCS, which allows for personalization of SCS based on real-time feedback, there is increased interest in personalized medicine in SCS treatment. Closed-loop integration with artificial intelligence (AI) could improve the efficiency of analyzing SCS parameters for patients by providing real-time feedback, analysis, and adjustment [110]. AI can collect patient outcome data and SCS data, which is then analyzed to provide information on biomarkers that signal tolerance or other physiologic changes [111]. Based on this analysis, providers can use AI to optimize the SCS settings for the patient, which may include variables like frequency, intensity, and duration [112,113]. AI and SCS synergy is a promising path to optimizing SCS for individual patients to provide pain relief and support.

Furthermore, there is interest in expanding the use of SCS to other indications. Currently, SCS is indicated most in cases of failed back surgery syndrome, and is also used to treat peripheral ischemia, peripheral neuropathy, and angina pectoris [113]. Overall, there is growing interest in understanding whether SCS can be expanded to relieve patients with other pain-related disorders.

One indication of interest is visceral pain that originates from internal organs. A comparative study on patients experiencing chronic abdominal pain found that those treated with SCS had better results in pain relief and decreased medication usage compared to patients treated with radiofrequency ablation of splanchnic nerves [11]. However, the evidence is limited, and more follow-up studies are needed to confirm the efficacy of SCS in visceral pain relief. A second indication of interest is spinal cord injuries. Current evidence is limited to case reports that found that SCS may be effective for pain relief in patients with spinal cord injury [114]. While the preliminary results are promising, there remains a need to conduct high-quality studies on a larger scale to test SCS effectiveness in treating spinal cord injury.

Additionally, there is interest in researching combination therapies with SCS further. A meta-analysis found that patients who were in the SCS treatment group had greater odds of reducing opioid use compared to those in the medical therapy group [115]. Thus, there is support that SCS may provide pain relief and reduce the amount of pain medication needed. Furthermore, there are case studies on combining SCS with peripheral nerve stimulation. These patients found that combined therapy provided greater relief than stimulation alone [116].

## 8. Conclusions

SCS emerged from the gate-control framework and evolved into complementary strategies to modulate pain levels. The current evidence base for SCS supports meaningful pain relief for patients. Despite innovations in providing stimulus without paresthesia, improvement in coverage, and multi-modal techniques, challenges in mediating inter-individual variability and difficulty isolating mechanisms in vivo persist. Further research elucidating mechanisms for paradigms such as high-frequency SCS will guide future studies and practice. There is considerable potential for personalized SCS therapy through AI-assisted programming, expansion of SCS to additional clinical indications, and the development of combination neuromodulation strategies to improve patient outcomes.

Despite advances in providing paresthesia-free stimulation, improving neural targeting, and developing adaptive programming strategies, the precise mechanisms underlying many SCS paradigms remain only partially understood. In particular, the relative contributions of spinal, supraspinal, neuroimmune, and molecular pathways warrant further investigation. Improved mechanistic understanding will facilitate the identification of predictive biomarkers, optimization of paradigms, and development of more personalized neuromodulation strategies.

## Figures and Tables

**Table 1:** Summarization of the key categorical pain mechanisms and the major elements and roles involved.

Category	Major Elements	Key Role in Pain
Glutamatergic Transmission	Glutamate (Aδ, C fibers), VGLUT2, NMDA and AMPA receptors, EAATs	Primary excitatory nociceptive signaling; EAATs prevent excitotoxicity
Presynaptic/Extrasynaptic Modulation	mGluRs (inhibitory or excitatory), endocannibinoids (CB1R), inhibitory interneurons (GABA, Glycine), opioids, ATP, descending monoamines (NE, DA, 5-HT)	Fine-tunes glutamate release and postsynaptic responses; balance of inhibition vs. facilitation shapes pain perception
Glial and Inflammatory Contribution	Pro-inflammatory cytokines (IL-1β, IL-6) Microglia (early), astrocytes (maintenance),	Drive and sustain neuropathic pain through inflammation and excitability
Central Sensitization	Increased glutamatergic transmission, NMDA/AMPA upregulation, ion channel changes, glial activation, LTP	Maladaptive plasticity leads to hyperalgesia and allodynia

**Table 2:** Tonic Spinal Cord Stimulation.

Primary Target/Cascade	Proposed Mechanistic Role
Aβ fibers → inhibitory interneurons/WDR	Segmental gate control: Aβ activation increases inhibitory drive, suppressing WDR neuron firing and nociceptive transmission.
GABAergic and glycinergic interneurons	Enhances inhibitory tone in dorsal horn, counteracting hyperexcitability in neuropathic states.
KCC2/Cl^−^ gradient	Partial restoration of impaired Cl^−^ homeostasis, improving GABAergic inhibition in injured dorsal horn.
Glutamatergic transmission (NMDA/AMPA, NR2B)	Reduces NMDA/AMPA activity and receptor phosphorylation, decreasing central sensitization.
Glial activation (microglial p38-MAPK, TLR4/NF-κB)	Attenuates microglial activation and pro-inflammatory signaling pathways in dorsal horn.
Cytokine profile (↓ IL-1β/IL-6/TNF-α; ↑ IL-10)	Shifts spinal milieu from pro- to anti-inflammatory, supporting long-term analgesia.
EAAT/GLT-1 and glutamate clearance	Improves astrocytic glutamate uptake, reducing excitotoxic drive onto dorsal horn neurons.

**Table 3:** High-frequency Spinal Cord Stimulation.

Primary Target/Cascade	Proposed Mechanistic Role
Aβ fibers/medium–small fibers (competing hypotheses)	Either depolarization block of Aβ fibers, preferential inhibition via interneurons, or modulation of nociceptive fibers.
Dorsal horn inhibitory interneurons (GABA/Gly)	Increases inhibitory interneuron output without conscious paresthesia, suppressing nociceptive transmission.
Kinase signaling (e.g., receptor-associated protein kinases)	Reduces kinase-dependent phosphorylation of NMDA/AMPA (incl. NR2B), dampening central sensitization.

**Table 4:** Burst Spinal Cord Stimulation.

Primary Target/Cascade	Proposed Mechanistic Role
Aβ-interneurons/WDR (segmental pathways)	Engages classical gate-control mechanisms with distinct burst timing, reducing WDR firing and nociceptive inflow.
GABA/Gly, KCC2, NMDA/AMPA (spinal excitability balance)	Modulates inhibitory/excitatory balance and Cl^−^ gradients similar to tonic SCS, but with burst-pattern input.
Supraspinal medial pain pathways (thalamo-cingulate, limbic)	Preferentially modulates affective–emotional pain processing via medial thalamus, ACC, and limbic circuits.

**Table 5:** Closed-loop Spinal Cord Stimulation.

Primary Target/Cascade	Proposed Mechanistic Role
ECAP amplitude as “tissue dose” proxy	ECAP magnitude reflects recruitment of dorsal column Aβ fibers; target ECAP window defines desired neural activation range.
Aβ-interneurons/WDR and associated spinal circuits	Same core cellular targets as tonic SCS, but with dynamic control to maintain consistent activation across physiologic changes.
Compensation for periprosthetic fibrosis and impedance changes	PID controller adjusts current to maintain ECAP within target window despite fibrosis, encapsulation, and electrode–tissue changes.

**Table 6:** A summarization of the mentioned SCS types and key information regarding their specific parameters and differences.

Modality	Stimulation Parameters	Proposed Mechanism of Action	Clinical Features	Limitations
Tonic SCS	10–500 Hz, 30–500 μs constant pulses	Activates Aβ fibers in dorsal horn → closes gate to nociceptive transmission (Gate Control Theory)	Produces paresthesia (“pins and needles”); well-studied; ~50–70% achieve >50% pain reduction	Limited relief in lower extremities/groin; uncomfortable paresthesia; habituation (loss of effect over time)
High Frequency SCS	Continuous 10 kHz stimulation	Paresthesia-free; thought to act via inhibitory interneurons in dorsal horn; possible modulation of medium/small fibers, interference with depolarization, inhibition of protein kinases	Strong efficacy (e.g., RCTs in diabetic neuropathy); patient preference due to no paresthesia	Mechanism unclear; long-term data limited
Burst SCS	Delivered in clusters (e.g., 5 pulses at 500 Hz)	Mimics natural neuronal burst firing; engages medial thalamo-cortical pathways (emotional/affective pain) in addition to sensory pathways	Effective for back and limb pain; no paresthesia; improves psychometric outcomes (mood/emotional state)	Mechanism not fully established; long-term comparative data still emerging
Closed-Loop SCS	Real-time ECAP-guided output adjustments	Uses feedback (ECAPs) to maintain neural activation within target window; ensures consistent tissue activation	Reduces variability, mitigates habituation; superior to open-loop for back/leg pain	Requires more complex technology; cost/availability concerns

**Table 7:** Mechanistic evidence supporting spinal cord stimulation paradigms by celluluar/molecular targets and level of evidence.

Paradigm	Supporting Article(s)	Cellular/Molecular Targets	Supporting Evidence	Level of Evidence
Tonic SCS	Bordeleau et al. [49]; Shealy et al. [5]	Dorsal column fibers, large-diameter Aβ afferents, dorsal horn gating circuits	Tonic SCS is supported by clinical sensory studies and early clinical reports showing that dorsal column stimulation can reduce pain through segmental modulation of nociceptive transmission.	Clinical
Tonic SCS	Joosten and Franken [50]; Smits et al. [51]	Aβ fibers, inhibitory interneurons, WDR neurons, GABA/glycine signaling, dorsal horn excitability	Mechanistic reviews and experimental SCS studies support the concept that tonic SCS activates non-nociceptive afferents and enhances inhibitory dorsal horn signaling, thereby suppressing WDR neuron activity and nociceptive transmission.	Mixed: preclinical and clinical
Tonic SCS	Patil et al. [52]	Neural adaptation pathways, habituation-related mechanisms	Habituation literature suggests that repeated stimulation may lead to reduced therapeutic response over time, supporting the need for alternative waveforms or adaptive stimulation strategies.	Review/mixed clinical-mechanistic evidence
High-frequency SCS	Abraham et al. [55]; Peeters and Raftopoulos [56]	Paresthesia-free dorsal horn modulation, possible nontraditional fiber recruitment	Clinical literature suggests that 10 kHz SCS provides analgesia without paresthesia, implying mechanisms beyond classic sensory masking by Aβ fiber activation alone.	Clinical/review
High-frequency SCS	Tieppo Francio et al. [57]	Inhibitory interneurons, dorsal horn excitability, small/medium fiber modulation, neuroinflammatory signaling	Summary of preclinical and clinical findings supports multiple possible mechanisms for 10 kHz SCS, including suppression of dorsal horn hyperexcitability and modulation of inflammatory or excitatory signaling.	Mixed: preclinical and clinical
High-frequency SCS	Petersen et al. [62]	Clinical pain pathway modulation in painful diabetic neuropathy	Randomized clinical trial evidence showed significant pain reduction with 10 kHz SCS plus conventional medical management compared with conventional medical management alone. This supports clinical efficacy, although it does not directly isolate the molecular mechanism.	Clinical RCT
High-frequency SCS	Arle et al. [58]	Dorsal column axons, depolarization-related mechanisms, paresthesia-free neural activation	Mechanistic modeling/theoretical work proposes that high-frequency stimulation may alter dorsal column axonal signaling and produce analgesia without conscious paresthesia.	Computational/mechanistic
High-frequency SCS	Liao et al. [59]	MAPK signaling, kinase activation, neuropathic pain-related dorsal horn pathways	Rat spared nerve injury data showed that early high-frequency SCS inhibited spinal MAPK activation and reduced neuropathic pain behavior, supporting a molecular anti-sensitization mechanism.	Preclinical
Burst SCS	Chakravarthy et al. [64]; Hou et al. [67]	Burst-pattern neural signaling, segmental and supraspinal pain pathways	Systematic and pooled clinical evidence supports burst SCS efficacy for chronic back and limb pain, suggesting that burst-pattern stimulation can provide analgesia without relying solely on tonic paresthesia-based masking.	Clinical systematic review
Burst SCS	Swadlow and Gusev [65]	Thalamocortical burst signaling, cortical activation	Basic neurophysiology evidence shows that burst firing can strongly influence cortical signaling, providing biologic plausibility for burst SCS effects on supraspinal pain processing.	Preclinical/basic neurophysiology
Burst SCS	Deer et al. [66]	Burst waveform effects on pain perception and patient-reported outcomes	The SUNBURST randomized controlled trial supports clinical efficacy of burst SCS and suggests benefit beyond traditional tonic stimulation, including paresthesia-free analgesia.	Clinical RCT
Burst SCS	Chakravarthy et al. [68]; Bocci et al. [69]	Medial thalamo-cortical pathways, affective-emotional pain networks, cortical plasticity	Mechanistic and neurophysiologic evidence suggests burst SCS may modulate both sensory-discriminative and affective-emotional pain pathways, including supraspinal/cortical mechanisms.	Mixed: clinical neurophysiology and review
Closed-loop SCS	Mangano et al. [70]; Zheng et al. [71]	ECAP-guided feedback control, dorsal column activation, adaptive stimulation control	Review-level evidence supports closed-loop SCS as an adaptive control strategy that uses real-time neural feedback to maintain more consistent spinal cord activation than open-loop systems.	Clinical/review
Closed-loop SCS	Versantvoort et al. [72]	ECAP-controlled stimulation, neuropathic pain circuitry, dorsal column recruitment	Experimental rat model evidence supports the feasibility of ECAP-controlled closed-loop SCS in neuropathic pain and provides preclinical support for ECAP-based feedback mechanisms.	Preclinical
Closed-loop SCS	Anaya et al. [73]; Parker et al. [75]	ECAP generation, dorsal column Aβ fiber recruitment, compound action potentials	Computational and human recording studies show that ECAPs reflect summed neural responses during SCS, supporting their use as a biomarker of dorsal column activation.	Mixed: computational and clinical neurophysiology
Closed-loop SCS	Mekhail et al. [74,76]	ECAP-controlled stimulation, neural activation consistency, clinical pain outcomes	EVOKE randomized clinical trial data and follow-up analyses show durable pain relief and improved outcomes with closed-loop SCS compared with open-loop SCS.	Clinical RCT and secondary clinical analysis
Closed-loop SCS	Ladner et al. [78]; Brucker-Hahn et al. [77,82]; Zander et al. [79] North et al. [80]; Pilitsis et al. [81]	ECAP amplitude/morphology, pulse width, posture, impedance, electrode-spinal cord distance, fiber recruitment	Preclinical, computational, and clinical neurophysiology studies show that ECAP morphology and neural activation vary with stimulation parameters, posture, and anatomy. These studies support ECAP feedback while also showing that constant ECAP amplitude may not always equal identical fiber recruitment.	Mixed: preclinical, computational, and clinical neurophysiology

**Table 8:** Animal Model connection to Human Studies and Clinical Trials.

Model/Target	In Vivo Effect	Confirmations in Humans	Status/Gap
Neuropathic pain models (CRPS-like, PSPS-like, peripheral neuropathy); dorsal horn cytokines (IL-1β, IL-6, TNF-α, IL-10)	SCS reduces IL-1β/IL-6/TNF-α and increases IL-10 in spinal cord/CSF; associated with behavioral analgesia	CSF and sometimes serum samples in CRPS, PSPS, and neuropathic patients show reduced IL-1β/IL-6 under SCS with clinical pain relief	Partial confirmation; small cohorts and heterogeneous sampling; need longitudinal, modality-specific cytokine profiling.
Dorsal horn WDR neurons and inhibitory interneurons (GABA/Gly, KCC2/Cl^−^ gradient)	SCS decreases WDR firing, restores inhibitory tone, and partially normalizes Cl^−^ homeostasis in dorsal horn	Indirect evidence via changes in pain thresholds, evoked potentials, and clinical analgesia under SCS	Strong mechanistic support in animals; lack of direct single-unit or KCC2 measurements in humans.
Glial activation (microglia/astrocytes; p38-MAPK, TLR4/NF-κB)	SCS reduces microglial activation markers and dampens pro-inflammatory signaling cascades in spinal cord	Limited CSF/serum inflammatory panels and occasional imaging/biopsy data suggesting decreased neuroinflammation	Convergent trend toward anti-inflammatory effects, but very limited human histologic or imaging confirmation.
Glutamate handling (NMDA/AMPA subtypes, NR2B; EAAT/GLT-1)	SCS decreases NMDA/AMPA phosphorylation, enhances glutamate uptake, and reduces excitatory drive	Human evidence largely indirect (improved pain and function; occasional MR spectroscopy data in related neuromodulation contexts)	Mechanism well-supported in rodent models; direct receptor or transporter measurements under SCS in humans are lacking.
Endocannabinoid system and CB1R signaling	Some preclinical SCS paradigms modulate spinal endocannabinoid tone and CB1R-mediated inhibition	Minimal direct data; endocannabinoid levels under clinical SCS rarely measured	Mechanistic hypothesis based mainly on animal work; human validation essentially absent.
SCI models (contusion, compression, traction, photochemical, ischemia-reperfusion) with SCS	SCS improves locomotor function, reduces secondary injury, and modulates inflammation and excitability	Observational data in SCI patients using SCS for pain and motor recovery show functional gains	General functional translation is encouraging, but molecular endpoints are poorly aligned between animal SCI models and clinical SCS cohorts.
ECAP-based closed-loop models (Aβ fiber recruitment)	Defined relationship between ECAP amplitude and recruited dorsal column fibers; improved stability of neural activation	Intraoperative and chronic ECAP recordings show that maintaining ECAP within a target window correlates with more stable analgesia	Strong biophysical and signal-level translation; gap remains in linking ECAP control to specific molecular and cellular changes in humans.

## Data Availability

No new data were created or analyzed in this study. Data sharing is not applicable to this article.
